# Editorial: The Neurobiology and Genetics of Gilles de la Tourette Syndrome: New Avenues through Large-Scale Collaborative Projects

**DOI:** 10.3389/fpsyt.2017.00197

**Published:** 2017-10-11

**Authors:** Peristera Paschou, Kirsten Müller-Vahl

**Affiliations:** ^1^Department of Biological Sciences, Purdue University, West Lafayette, IN, United States; ^2^Department of Psychiatry, Socialpsychiatry and Psychotherapy, Hannover Medical School, Hannover, Germany

**Keywords:** Gilles de la Tourette syndrome, genetics, neurobiology, treatment, clinical research, collaborative studies

**Editorial on the Research Topic**

**The Neurobiology and Genetics of Gilles de la Tourette Syndrome: New Avenues Through Large-Scale Collaborative Projects**

Gilles de la Tourette syndrome (TS) is a childhood-onset neurodevelopmental disorder with an estimated prevalence of 0.3–0.9% (1, 2). Although the occurrence of multiple motor and vocal tics is key to the diagnosis, the clinical phenotype is extremely heterogeneous with only 10–13.5% of pure TS cases (i.e., tics only), and the vast majority of patients presenting with additional psychiatric comorbidities (3–5). For instance, TS is commonly associated with comorbid attention-deficit hyperactivity disorder (ADHD, in about 60% of patients), obsessive-compulsive disorder (OCD, in 30–50% of patients) and to a lesser extent depression, anxiety, autism spectrum disorder (ASD), and others (3, 6–8). There is no cure for TS and treatment aims to only alleviate symptoms. Our search for novel therapies that may significantly improve patient quality of life is hampered by our limited understanding of the pathophysiology of the disorder. A complex and still unclarified genetic background further modified by non-genetic factors, such as infections, autoimmunity, neural, and psychosocial stressors, is implicated in TS pathogenesis (1, 9). Parallel, interacting cortico-striato-thalamo-cortical (CSTC) circuits, linking specific regions in the frontal cortex to subcortical structures (including the basal ganglia and thalamus) are thought to be involved together with abnormalities in the dopamine, glutamate, serotonin, histamine, and acetylcholine systems (1).

For a very long time, efforts to elucidate the etiology and pathophysiology of TS have been fragmented and hampered by low statistical power. Finally, after decades of active research aiming to identify the etiology and pathophysiology of TS, we are on the verge of a new era, promising exciting and rapid discoveries in the field. Investigators from around the world, representing multiple disciplines and scientific approaches, are joining their efforts in large-scale initiatives and multiple resources are being consolidated and coming together to serve the study of TS, including large well-characterized patient cohorts, specialized epidemiological databases, and novel analytical tools that allow integrated systems biology approaches. These are supported both by European Union and US National funding agencies, as well as patient support and advocacy groups such as the Tourette Association of America and Tourette’s Action UK. This Research Topic was motivated by large-scale initiatives, such as the Marie Curie Initial Training Network TS-EUROTRAIN (Forde et al.), the rapid growth of the European Society for the Study of Tourette Syndrome (10, Mathews and Stern et al.), and an important milestone in TS international research collaboration, the First World Congress on Tourette Syndrome and Tic Disorders (Mathews and Stern et al.). We reached out to the whole of the TS research community in order to put together a special issue that showcases current large-scale efforts in the field, while covering both clinical and etiological aspects of TS and providing an excellent overview about current knowledge and areas of research in this complex neurodevelopmental disorder.

TS represents a model complex disorder with great clinical heterogeneity pointing to an equally complex and heterogeneous etiological basis. Thus, understanding the clinical spectrum of the disorder is the first step toward improved patient management but also uncovering the pathophysiology of the disorder. In this issue, new insights into clinical characteristics of TS are presented by Sambrani et al., who analyzed clinical data in 1,032 patients with TS from a single center. The results give clinically relevant new information about tics, premonitory urges, and comorbidities. Tics are typically preceded by premonitory urges, but until today the relation between tics and urges is not understood. Therefore, it is important to have reliable assessments for both tics and premonitory urges. Brandt et al. investigated the validity of the “Premonitory Urge for Tic Disorders Scale” (PUTS) and suggest to develop different subscales of the PUTS, since there is evidence for more than one dimension of urges in patients with TS. Ruhrman et al. specifically focus on “non-motor aspects” of TS, including tic-related cognitions, the influence of environmental factors on tics and sensory modulation disorder. Recent studies and clinical experience suggest that stress often worsens tics. Buse et al. report results from an experimental study investigating the effect of stress on tics in children with TS and interestingly found that stress resulted in a situational decrease of tic frequency. Eapen et al. summarized available data on quality of life in patients with TS and highlight the social impact of the disease on both an individual’s and family’s life. An under-recognized symptom that may impair in particular children’s health related quality of life is described by Zanaboni Dina et al. They point out that handwriting is one of the most impaired school activities in children with TS and report about a case with severe “handwriting tics.” Robinson et al. describe the phenomenon of “tic attacks” in patients with TS and discuss the etiology of this clearly underreported symptom. They suggest that “tic attacks” resemble a combination of tics and functional movements and give recommendations for the treatment of “tic attacks.”

Still on the clinical front, the optimal treatment strategy for TS patients must take into consideration tic severity as well as determine which co-existing symptoms are the most prominent, disabling and causing the patient the most difficulty (1). Behavioral interventions are currently considered the first-line treatment for tics (11–14). However, the limited number of trained therapists, inconveniences such as travel distance, and willingness to engage can serve as barriers. Here, Jakubovski et al. alternatively suggest a sophisticated internet-delivered treatment program for comprehensive behavioral intervention for tics (CBIT) and describe the protocol of a large randomized controlled trial (ONLINE-TICS). Morand-Beaulieu et al. used a modified CBIT program, called the cognitive–psychophysiological (CoPs) model, to treat patients with both TS and body-focused repetitive behaviors (BFRB). They found that CoPs improves both types of symptoms and suggest that CoPs therapy modifies attentional processes as demonstrated by altered event-related potentials (ERP). Leclerc et al. suggest “Facotik therapy” as an alternative treatment for tics: Facotik was adapted from the adult cognitive and psychophysiological program for tics. The authors present data suggesting that Facotik therapy may be effective in tic reduction in children with TS due to a modification of cognitive–behavioral and physiological processes.

Pharmacological interventions are typical second-line options whereas experimental approaches include deep brain stimulation (DBS) for severe and treatment refractory cases (1). In an open-label uncontrolled study, Gerasch et al. were able to demonstrate that aripiprazole improves not only tics but also OCD and possibly other comorbidities, including depression, anxiety, and ADHD, but has no influence on premonitory urges. Surgical treatment with DBS has been suggested as a promising therapy in otherwise treatment-resistant patients with TS. Pedroarena-Leal and Ruge summarized all available data on both invasive and non-invasive stimulation techniques for TS and, in addition, discuss novel applications for neurostimulation techniques based on a symptom-guided approach. Since the database on DBS in TS is still weak, it was very important to build up a DBS database to further increase our knowledge about efficacy and safety of DBS in TS. Deeb et al. give an excellent overview on this international DBS registry and explain how it works. Haense et al. used single photon emission computed tomography (SPECT) and 99mTc-ECD to investigate the effects of DBS in both globus pallidus internus (GPi) and centromedian-parafascicular/ventralis oralis internus nuclei of the thalamus (CM/Voi) and sham stimulation on cerebral blood flow. They found altered brain perfusion in the frontal cortex and the cerebellum that can be reversed by both GPi and CM/Voi DBS. Finally, Jimenez-Shahed et al. recorded intraoperative local field potentials (LFPs) from the postero-ventrolateral GPi in unmedicated Parkinson’s disease (PD) patients and patients with TS (both at rest, during voluntary movements, and during tic activity). From their data, it is suggested that beta-high frequency oscillations (HFO) cross-frequency coupling (CFC) in the GPi might be specific to involuntary movements in general.

Neuroimaging studies may uncover clues to the complex pathways and brain circuits underlying TS, although to-date studies are limited by small sample size. In a subset of papers in this issue, results from neuropsychological and neuroimaging studies are reported. Eichele et al. used a task measuring performance monitoring and found that children with TS may employ additional attentional resources as a compensatory mechanism to maintain equal behavioral performance. In two other papers, data from neuroimaging studies are reported in patients with common comorbidities in TS, OCD, and ADHD. Fan et al. used diffusion tensor imaging (DTI) and investigated both patients with OCD, unaffected siblings, and healthy controls and found white matter alterations in the left cingulum bundle in OCD, which were partly also seen in unaffected siblings. In patients suffering from ADHD, Forde et al. found no changes in cortical gyrification or intrinsic curvature compared to healthy controls. It is worth noting that large-scale neuroimaging studies for neuropsychiatric disorders are now starting to emerge [see, for instance, Ref. (15, 16)]. However, we are only just entering the era of such large-scale neuroimaging studies in TS and efforts such as the newly established ENIGMA-TS working group will undoubtedly prove pivotal to increasing our understanding of the neurophysiology of TS and the link between brain circuits and genetic background (http://enigma.ini.usc.edu/ongoing/enigma-ts/).

Indeed, several twin and family studies have demonstrated that TS is one of the most heritable, non-Mendelian neuropsychiatric disorders with the population-based heritability estimate estimated at 0.77 (17–19). However, to date no definitive TS-associated risk gene of major effect has been identified [Georgitsi et al.; (9)] although recent large-scale studies have provided evidence for the first robust genetic associations to the disorder (20, 21). These landmark discoveries were made possible thanks to international collaboration. Here, Georgitsi et al. offer a comparative report of active large-scale efforts aiming to understand the genetic etiology of TS, including the Tourette Syndrome Association International Consortium for Genetics (TSAICG), TIC Genetics targets rare, the European Multicentre Tics in Children Study (EMTICS), and TS-EUROTRAIN, a Marie Curie Initial Training Network. Each of these initiatives represents a range of different approaches to the study of disorders with complex inheritance; from genome-wide association studies targeting common variants to exome sequencing for rare variants and integration with neurophysiological and gene-expression findings. Importantly, these complementary large-scale efforts are joining forces to uncover the full range of genetic variation and environmental risk factors for TS, holding great promise for identifying definitive TS susceptibility genes. In this issue, we also present studies that follow-up on promising leads for TS genetics [Alexander et al.; Padmanabhuni et al.] and include a critical review of the functional evaluation of genes that have been previously found to be disrupted in TS patients (Sun et al.). The gap between gene identification and underlying biology still remains to be bridged.

The high comorbidity rates with other neurodevelopmental disorders, such as OCD, ADHD, and ASD, lend support to the hypothesis of a shared etiological basis and suggest that genes underlying TS susceptibility actually have a role across neurodevelopmental phenotypes (22). Thus, TS can be considered a model disorder that can help shed light into the etiology of other neurodevelopmental disorders as well. Indeed, family studies indicate that, within TS families, OC symptoms and tics are etiologically related (23), while ADHD symptoms have also been shown to be etiologically related although in a more complex manner (24). Cross-disorder analysis may indeed provide clues to such shared etiological basis. In this issue, Tsetsos et al. present the first meta-analysis of GWAS for TS and ADHD and offer support for a shared etiological basis. In a large-scale study, including participants in the Netherlands Twin Register, Zilhão et al. find substantial genetic correlations between hoarding, OC symptoms, and tics.

Consistent with observations of other neurodevelopmental disorders, increasing evidence links neural and immune interactions to the pathogenesis of TS (1). For instance, streptococcal infection has been implicated as an environmental trigger leading to TS onset (25). Here, Spinello et al. critically discuss the available evidence in preclinical models in support of the link between TS and pediatric autoimmune neuropsychiatric disorders associated with streptococcus infections (PANDAS), as well as the limitations of these studies. Intriguingly, a case report included in this collection also suggests a relationship between *S. aureus* colonization and tic improvement (Eftimiadi et al.). The link between immunity and TS pathophysiology still remains to be fully explored.

Epigenetic mechanisms may mediate the effect of environmental triggers on genetic background, thus leading to the onset of TS. Pagliaroli et al. provide a summary of the recent findings in genetic background of TS, followed by an overview on different epigenetic mechanisms, such as DNA methylation, histone modifications, and non-coding RNAs in the regulation of gene expression. Epigenetic studies in other neurological and psychiatric disorders are discussed along with the TS-related epigenetic findings available in the literature to date. Moreover, they offer evidence that some general epigenetic mechanisms seen in other neuropsychiatric disorders may also play a role in the pathogenesis of TS.

Animal models of tics could help elucidate the complex interplay between genetic, environmental, and neuroimmunological risk factors, and facilitate the development of improved therapies. However, still considerable debate exists over the validation of TS animal models. Here two comprehensive reviews (Nespoli et al.; Yael et al.) present all existing TS models highlighting recent advances as well as the need to overcome shortcomings. Importantly, Yael et al. call for a standardization process in the study of TS animal models as the next logical step. They suggest that a generation of standard examination criteria will improve the utility of these models and enable their consolidation into a general framework. This should lead to a better understanding of these models and their relationship to TS, thereby improving the research of the mechanism underlying this disorder and aiding the development of new treatments.

Thanks to international collaboration, we are on the verge of a new era promising exciting discoveries on the neurobiology of TS. For instance, large well-characterized cohorts of TS patients have become available, and US and European TS genetics consortia have harmonized phenotypic assessments and established pre-publication data sharing and joint meta-analyses [Georgitsi et al.; (26)]. As a result, already the first definitive TS risk genes have been identified although they still encompass a small portion of the overall TS susceptibility risk (20, 21). The next step will now be to shift from linear thinking to more complex, integrated and multi-dimensional approaches (Lessov-Schlaggar et al.). TS is not a unitary condition and as such patients also respond to treatment in different ways. This highlights the importance of thinking across diagnostic categories when attempting to understand the neurobiology of these phenotypes (27–29). The development of quantitative TS phenotypes and analyzing across a spectrum rather than on ends of a distribution may hold the promise to unravel the etiology of TS and be the starting point to personalized medicine in TS.

## Author Contributions

PP coordinated the Research Topic and wrote the manuscript. KM-V coordinated the Research Topic and wrote the manuscript.

## Conflict of Interest Statement

The authors declare that the research was conducted in the absence of any commercial or financial relationships that could be construed as a potential conflict of interest.
